# Cyclophilin D (PPIF) and MPTP in hepatic ischemia-reperfusion injury: insights into mechanisms

**DOI:** 10.3389/fimmu.2025.1575242

**Published:** 2025-08-29

**Authors:** Jingxin Liu, Chengyu Wu, Ziyun Lin, Maomao Ma, Wei Ma, Xuefeng Yu, Kai Wang, Bin Zeng

**Affiliations:** ^1^ College of Pharmacy, Shenzhen Technology University, Shenzhen, China; ^2^ Department of Biotechnology Research and Development, Guangzhou Dihe Biotechnology Co., Ltd, Guangzhou, China; ^3^ Research Center for Preclinical Medicine, Southwest Medical University, Luzhou, China

**Keywords:** cyclophilin D (CypD), MPTP, hepatic ischemia-reperfusion injury, mechanisms, mitophagy, inflammatary disease, calcium overload

## Abstract

Hepatic ischemia-reperfusion injury (HIRI) is a major complication in liver transplantation, hepatic surgeries, and shock-induced acute liver failure. This injury is characterized by mitochondrial dysfunction, oxidative stress, and calcium overload, with the mitochondrial permeability transition pore (mPTP) playing a pivotal role in mediating hepatocyte death. Cyclophilin D (CypD), a key regulator of mPTP opening, has long been associated with the exacerbation of HIRI. However, recent research has uncovered a protective aspect of CypD, revealing that it can regulate intermittent or “flickering” mPTP openings to control calcium overload, preserve mitochondrial integrity, and mitigate damage during ischemic stress. This review highlights the dual role of CypD in regulating mitochondrial damage through mPTP dynamics and its complex interplay with autophagy, specifically mitophagy, in liver injury. We also explore the emerging pharmacological and genetic approaches targeting PPIF, offering potential avenues for mitigating liver injury in clinical settings. This review integrates recent findings on PPIF’s role in mPTP regulation, inflammation, autophagy, and mitophagy, proposing a nuanced view of its therapeutic potential in managing hepatic ischemia-reperfusion injury.

## Introduction

Hepatic ischemia-reperfusion injury (HIRI) is a critical factor in multiple clinical scenarios, notably liver transplantation, hepatic surgeries, and shock-related acute liver failure. It is a major complication of hemorrhagic shock, liver resection, and transplantation, reflecting the dual insult of ischemia and inflammation-mediated reperfusion injury (1). Notably, HIRI is estimated to account for ~10% of early liver graft failures​ (2). It also heightens the risk of acute and chronic rejection of the liver graft​ (3), and has been implicated in long-term complications such as graft fibrosis and even cancer recurrence in transplant recipients​ (4). Severe ischemia-reperfusion damage can lead to primary non-function of the transplanted liver (5) and is a major risk factor for both acute and chronic rejection.

In hepatic surgery (e.g. liver resections), temporary inflow occlusion (the Pringle maneuver) or vascular clamping is often necessary to control bleeding, but it induces warm ischemia (6). Prolonged ischemia during major liver resections can result in significant HIRI, which in turn may cause postoperative liver dysfunction or even acute liver failure in susceptible patients​. Indeed, hepatic IRI is a frequent cause of acute liver failure in the perioperative period, especially after extensive liver resection or transplantation (7). The systemic consequences of severe HIRI are also clinically important: reperfusion injury to the liver can trigger a systemic inflammatory response and remote organ injury. For example, patients with major HIRI often develop acute kidney injury (AKI) as part of a multi-organ dysfunction syndrome (MODS), and the combination of liver injury with AKI dramatically increases perioperative morbidity and mortality​ (8–10). HIRI in some cases precipitates systemic inflammatory response syndrome (SIRS) or MODS, both of which carry high mortality (11, 12).

## Mitochondrial Dysfunction and the MPTP

Mitochondria are central executors of cell death in hepatic IRI. Ischemia deprives hepatocytes of oxygen, causing mitochondrial ATP production to plummet and leading to ionic imbalances (e.g. Ca^2+^ overload) within cells. Upon reperfusion, the combination of restored oxygen (fueling ROS generation) and normalization of pH creates the conditions for opening of the mitochondrial permeability transition pore (MPTP). The MPTP is a non-specific, high-conductance channel that can form in the inner mitochondrial membrane under stress. When the MPTP opens, it allows solutes <1.5 kDa to flood across the mitochondrial membranes, dissipating the proton gradient (13). Consequently, mitochondria depolarize (losing membrane potential) and halt ATP synthesis, and they often swell and rupture. MPTP opening also releases pro-apoptotic factors sequestered in mitochondria – notably cytochrome c and other proteins – into the cytosol (14). It is widely acknowledged that MPTP opening plays a crucial role in cell death after I/R injury, essentially serving as a point-of-no-return for stressed hepatocytes (15, 16).

**Figure 1:**
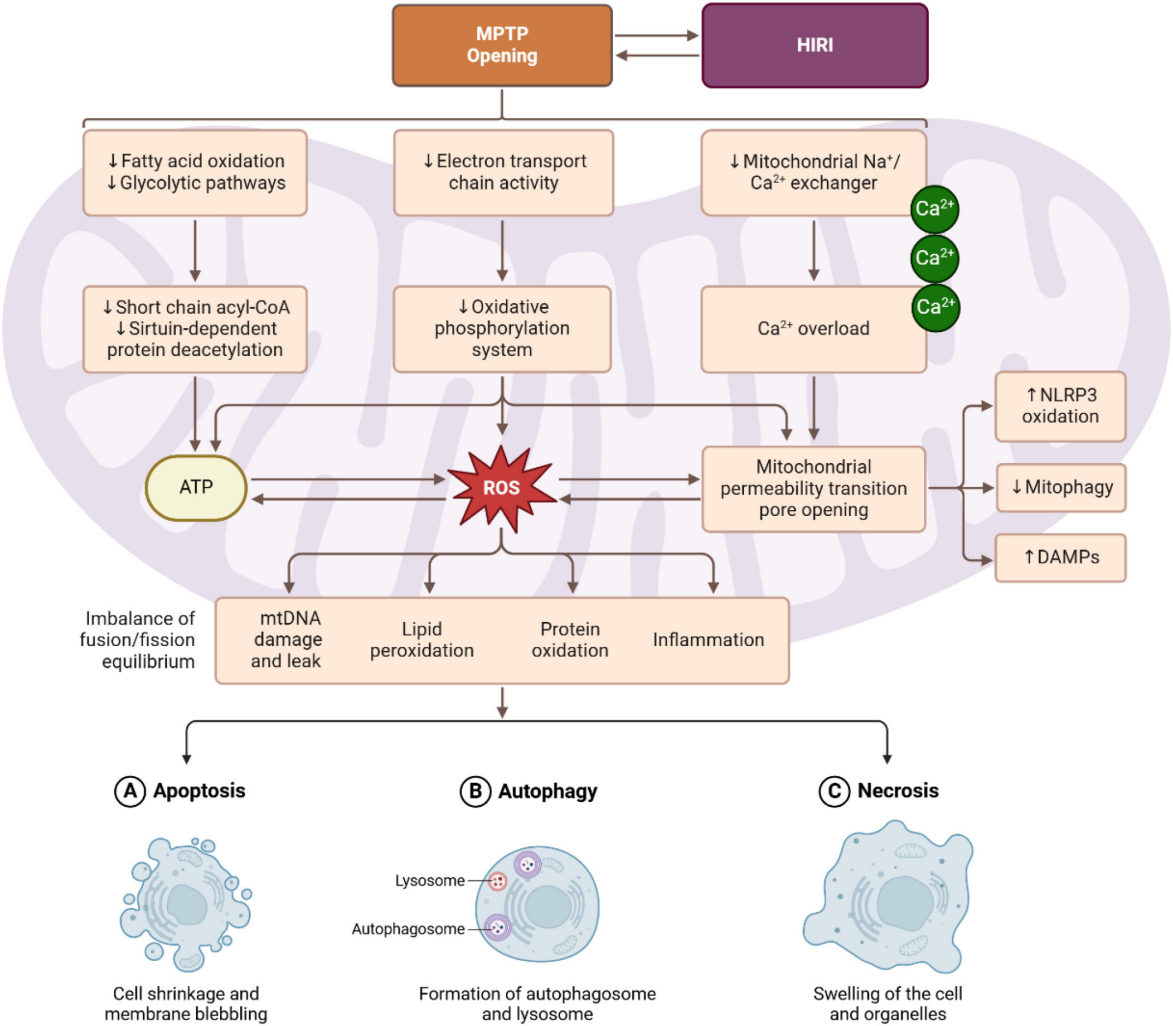
Overview of Hepatic Ischemia-Reperfusion Injury (HIRI) process.

Importantly, MPTP opening can lead to both apoptotic and necrotic cell death, depending on the extent and duration of pore opening (**Figure 1**). Transient or partial opening of the MPTP may predominantly trigger apoptosis by releasing cytochrome c to activate caspases, while allowing some mitochondria to remain functional. In contrast, sustained MPTP opening causes a collapse of mitochondrial energetics in the cell, resulting in profound ATP depletion and oncotic necrosis (17). In liver IRI, necrosis often co-exists with apoptosis; studies have shown that blocking MPTP tends to reduce necrosis extensively and can also reduce apoptosis. On the other hand, preventing MPTP opening preserves mitochondrial integrity—maintaining membrane potential and preventing cytochrome c release — thereby avoiding the apoptotic cascade​ (18).

## Central role of cyclophilin D in MPTP

A central regulator of the MPTP is Cyclophilin D, a mitochondrial peptidyl-prolyl isomerase that facilitates pore opening (19). Although the exact molecular composition of the mitochondrial permeability transition pore (MPTP) continues to be debated, mounting evidence suggests that it involves a multiprotein complex in the inner mitochondrial membrane (IMM), with key components including the FoF1-ATP synthase oligomer (20), the adenine nucleotide translocase (ANT) (21), and associated regulatory proteins such as Cyclophilin D (CypD). Early models proposed that ANT was the central pore-forming unit, whereas more recent work indicates that the c-ring of the FoF1-ATP synthase may form the channel component of the MPTP under pathological conditions (22, 23). Regardless of the exact structural arrangement, these protein complexes can transiently adopt a high-conductance state that renders the IMM permeable to solutes up to approximately 1.5 kDa in size.

**Figure 2:**
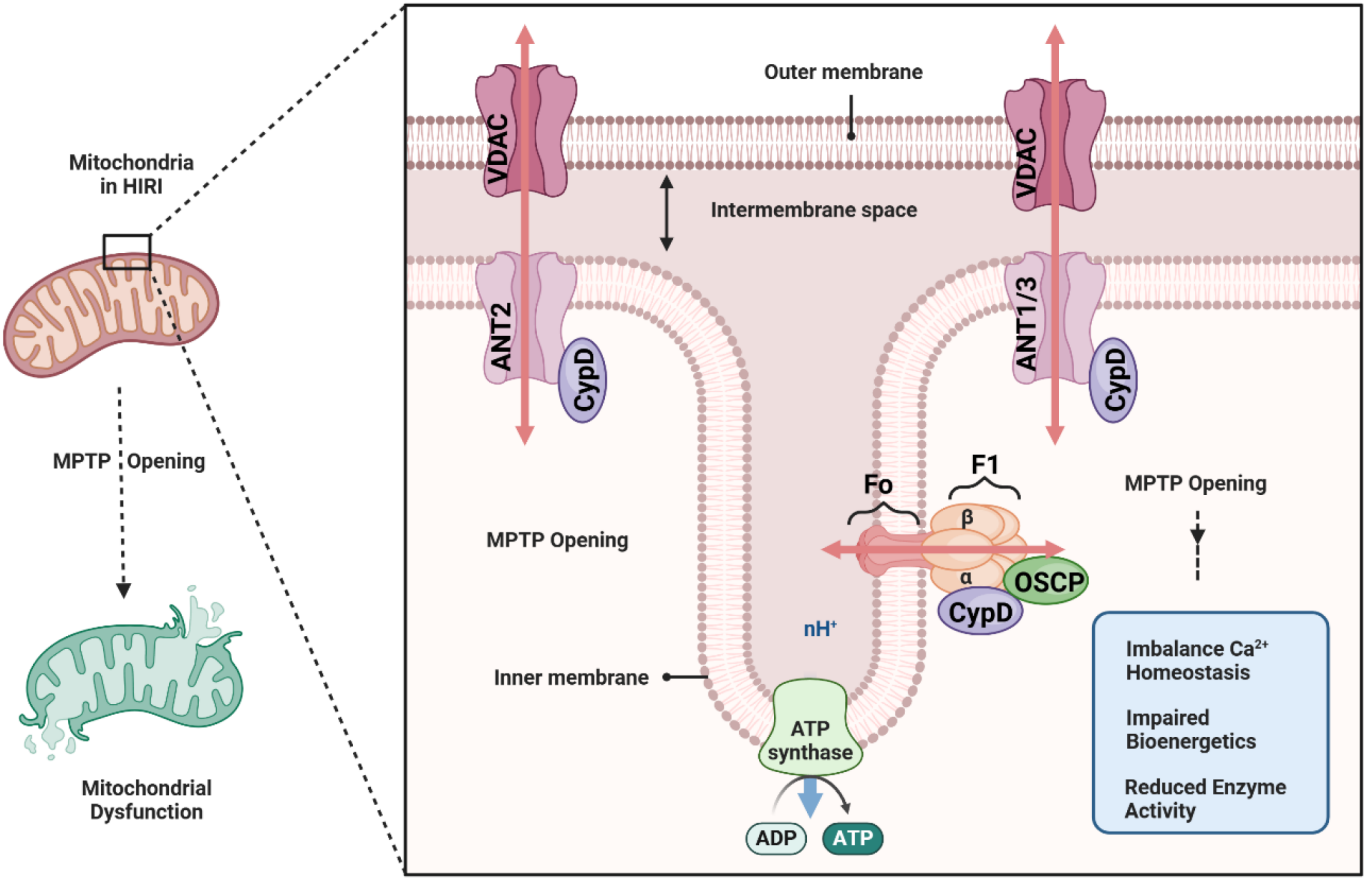
Mitochondrial Permeability Transition Pore (mPTP) opening mechanism.

CypD, encoded by the PPIF gene, is a peptidyl-prolyl isomerase located in the mitochondrial matrix that exerts a critical regulatory influence on the MPTP. Under normal physiological conditions, CypD remains loosely associated with its binding partners, having minimal impact on IMM permeability. However, in pathologic states—particularly those involving calcium overload and oxidative stress—CypD binds to the MPTP complex and lowers the threshold for pore opening. The structure and composition of the MPTP, though not fully resolved, converge on a final common pathway: when triggered by Ca^2+^overload and other stressors, this pore can catastrophically disrupt mitochondrial homeostasis. CypD occupies the critical position of “gatekeeper” for MPTP activation (24), making it a prime target for therapeutic strategies aimed at preserving mitochondrial integrity in hepatic ischemia-reperfusion injury and other mitochondrial-related pathologies (**Figure 2**).

Recent molecular studies using transgenic models have underscored the pivotal role of CypD in HIRI. Liver-specific CypD knockout mice are remarkably protected from ischemia-reperfusion damage: they show reduced hepatocellular necrosis, lower ROS levels, and decreased apoptosis compared to wild-type mice. In contrast, overexpression of CypD exacerbates mitochondrial injury and liver enzyme release after I/R (25). These findings identify the CypD-mediated MPTP opening as a linchpin event in HIRI, linking upstream stress signals (calcium overload, oxidative stress) to cell death outcomes (apoptosis/necrosis). Consistently, interventions that inhibit the MPTP have demonstrated potent hepatoprotective effects. The classic MPTP inhibitor cyclosporine A, which binds CypD, has been shown to significantly attenuate liver IRI – an observation documented for decades (26, 27)​. Overall, mitochondrial damage – and specifically MPTP opening – is a key mediator of hepatocellular injury in IRI, integrating the effects of calcium overload and ROS with the execution of cell death. Protecting mitochondrial integrity correlates with improved liver function and survival in models of HIRI, highlighting the role of CypD in preventing MPTP as a prime therapeutic target in ischemia-reperfusion injury of the liver.

The opening of mPTP not only results in energy collapse but also exacerbates oxidative stress. When the pore opens at reperfusion, electrons leak from the electron transport chain, generating bursts of ROS. The sudden influx of calcium and loss of mitochondrial control further impair metabolic enzymes, leading to a vicious cycle of ROS overproduction, PPIF’s role in mPTP means it indirectly governs this ROS surge: by facilitating pore opening, active CypD causes more oxidative damage, whereas PPIF inhibition limits ROS generation during reperfusion. ​Consistently, livers lacking CypD show significantly lower ROS levels after I/R​, highlighting how PPIF contributes to oxidative stress in HIRI (25).

PPIF-dependent mPTP opening can trigger both apoptotic and necrotic pathways of cell death in the liver. On one hand, prolonged mPTP opening causes outright necrosis due to catastrophic ATP loss – indeed, necrosis is a major form of cell death in hepatic I/R injury when mitochondria can no longer sustain viability (28). On the other hand, transient or partial pore openings induce apoptosis: mitochondrial swelling leads to outer membrane rupture and release of cytochrome c and other apoptogenic factors, activating caspases​. On the other hand, transient or partial pore openings induce apoptosis: mitochondrial swelling leads to outer membrane rupture and release of cytochrome c and other apoptogenic factors, activating caspases​. In a mouse liver I/R model, CypD deletion blocked the increase in cleaved caspase-3 and protected hepatocytes from apoptosis, whereas CypD overexpression heightened caspase-3 activation and apoptotic cell loss. Thus, PPIF-driven mPTP opening is a proximal event leading to both caspase-dependent apoptosis and energy-depletion necrosis in ischemia-reperfusion injury (28).

## Cyclophilin D (PPIF) and the activation of inflammatory

Reperfusion triggers abrupt calcium influx and oxidative stress, which induce MPTP opening and collapse the mitochondrial membrane potential​. This catastrophic bioenergetic failure leads to cell death, predominantly necrosis (and to some extent apoptosis), in the ischemic liver (29). Necrotic hepatocytes lose plasma membrane integrity and release their intracellular contents as damage-associated molecular patterns (DAMPs) (30, 31), which act as “danger” signals to the immune system (32). The aftermath of MPTP-mediated cell death is a burst of DAMPs from injured hepatocytes, which serve as inflammatory triggers. The aftermath of MPTP-mediated cell death is a burst of DAMPs from injured hepatocytes, which serve as inflammatory triggers. Circulating mitochondrial DAMPs have been identified as major mediators of systemic inflammation, capable of precipitating a SIRS-like response​ (33). Extracellular ATP is a potent DAMP released from necrotic cells. Excess ATP signals through purinergic P2X7 receptors on immune cells, triggering K^+^ efflux and activation of the NLRP3 inflammasome​ (34–36). In hepatic I/R, ATP released from dying hepatocytes acts as a “danger” signal that activates Kupffer cells and other immune cells, promoting IL-1β maturation and inflammatory cytokine release​. HMGB1 is another nuclear DNA-binding protein that is passively released by necrotic cells and actively secreted by stressed immune cells. It emerges early after hepatic reperfusion and is elevated in I/R-injured livers​ (37). Once extracellular, HMGB1 binds to pattern recognition receptors (such as TLR4 and RAGE) on Kupffer cells and endothelial cells, amplifying inflammation and recruiting leukocytes to the liver​. Once extracellular, HMGB1 binds to pattern recognition receptors (such as TLR4 and RAGE) on Kupffer cells and endothelial cells, amplifying inflammation and recruiting leukocytes to the liver​. Collectively, these DAMPs alert and activate the innate immune system in the liver. Kupffer cells (resident macrophages) detect the released mitochondrial and nuclear contents via their pattern recognition receptors, interpreting them as signs of danger​ (32).

When Cyclophilin D binds to the MPTP complex, it lowers the threshold for pore opening, thereby promoting mitochondrial swelling and dysfunction. In liver I/R, CypD-mediated MPTP opening greatly exacerbates cell injury: experiments show that liver-specific CypD knockout mice suffer far less necrosis and oxidative stress during I/R, whereas overexpression of CypD aggravates hepatocyte death (25). By encouraging pore opening, PPIF accelerates ATP depletion and reactive oxygen species (ROS) generation in hepatocytes, pushing cells toward necrotic and apoptotic death. The excess ROS produced can oxidize cellular components and act as secondary messengers that further amplify inflammation. In essence, Cyclophilin D serves as a pro-death switch – its activity tips stressed mitochondria into failure, and the resulting cell injury releases the inflammatory triggers described above (**Table 1**).

**Table 1:** Experimental interventions targeting cyclophilin d (CypD) and the MPTP in hepatic ischemia-reperfusion injury: mechanisms and protective outcomes.

No.	Study model	Mechanism of I/RI induction	Results	Mechanism	Target
1 (28)	mice	70% liver I/R injury with 60 min of ischemia and 4 h of reperfusion	inhibition of CypD protected against liver injury, reducing hepatocyte necrosis and apoptosis.	PPIF (CypD) is critical for MPTP-mediated mitochondrial dysfunction and hepatocyte death.	CypD
2 (25)	mice	70% liver I/R injury with 90 min of ischemia and 6 h of reperfusion	liver‐specific knockout of CypD alleviated necrosis and dysfunction	reducing apoptosis and autophagy through caspase‐3/Beclin1 crosstalk	CypD
3 (73)	rat	70% liver I/R injury with 60 min of ischemia and 6 h of reperfusion	EP4 agonist (CAY10598) alleviated necrosis and inflammatory responses;MPTP opener carboxyatractyloside and the ERK1/2 inhibitor PD98059 partially reversed the effects.	EP4 -ERK1/2-GSK3β axis induce MPTP inhibition to provide hepatoprotection	MPTP
4 (16)	mice	70% liver I/R injury with 60 min of ischemia and 8h of reperfusion	CypD knockout or inhibitor diminish MPTP opening, lead to calcium-induced mitochondrial swelling decrease.	CypD knockout or inhibitor diminish restored hepatic calcium retention capacity and oxidative phosphorylation parameters	CypD
5 (63)	rat	70% liver I/R injury with 60 min of ischemia and 4 h of reperfusion	Trisulfate Disaccharide diminished the intracellular calcium raise preserves mitochondrial function and increases hepatic tolerance to I/R injury.	Inhibiton of Trisulfated disaccharide (NCX), decreasing calcium overload to inhibit MPTP opening	MPTP
6 (72)	mice	liver I/R injury with 45 min of ischemia and 6 h of reperfusion	NIM811, a specific mPTP opening inhibitor, reduced Cell Deaths.	MPTP opening inhibition Reduced the imbalance of mitiochondrial homeostasis	MPTP
7 (17)	mice	70% liver I/R injury with 60 min of ischemia and 8h of reperfusion	c-Jun N-terminal kinase 2 (JNK2) knockout moderate MPTP opening protected against liver injury, reducing hepatocyte necrosis and apoptosis.	JNK2 mediated MPTP opening contributes to increased mitochondrial dysfunction	MPTP

Beyond simply causing cell death, CypD-driven MPTP opening has direct ramifications for inflammatory signaling. Uncontrolled pore opening leads to the release of mitochondrial constituents (mtDNA, etc.) and ROS into the cytosol and extracellular space, which immune cells recognize as danger signals. One major consequence is the activation of inflammasomes, specifically the NLRP3 inflammasome complex, in liver resident immune cells. NLRP3 Inflammasome Activation: Kupffer cells that internalize or encounter DAMPs get “primed” (via signals like HMGB1 or lipids binding TLRs) and “activated” (via signals like ATP and ROS) to assemble the NLRP3 inflammasome​ (34, 36). The efflux of K^+^ triggered by ATP and the presence of mitochondrial ROS/mtDNA are known activating signals for the NLRP3 inflammasome multiprotein complex. Upon assembly, NLRP3 recruits and activates caspase-1, which in turn cleaves pro–IL-1β and pro–IL-18 into their active, inflammatory forms. In hepatic I/R, this pathway is a key link between mitochondrial dysfunction and inflammation: the DAMPs originating from MPTP-induced cell injury drive inflammasome activation and pyroptotic signaling in Kupffer cells (38). As a result, active IL-1β and IL-18 are released, amplifying local inflammation. Tumor necrosis factor-α (TNF-α) and other cytokines are also induced as part of this cascade. Notably, studies have found that blocking Cyclophilin D or knocking out PPIF can indirectly dampen these immune responses – by preventing MPTP opening, there is less mitochondrial DAMP release and oxidative stress to trigger NLRP3 (39). Thus, CypD-dependent MPTP opening is not only a mediator of cell death but also a catalyst for innate immune activation in the reperfused liver.

## Role of autophagy and the involvement of CypD in HIRI

Autophagy, a highly regulated process of lysosomal self-digestion, plays a dual role in hepatic ischemia-reperfusion injury (HIRI). On the one hand, moderate or timely activation of autophagy is generally considered hepatoprotective, as it selectively removes damaged mitochondria (mitophagy) and other injured organelles that accumulate during ischemic stress. In HIRI, autophagy is activated as a response to cellular damage caused by ischemia, including mitochondrial dysfunction and oxidative stress. Studies have shown that young plasma, when administered to aged rats, can restore age-impaired autophagy and reduce liver injury by enhancing autophagic activity via the AMPK/ULK1 signaling pathway. This mechanism is crucial as AMPK phosphorylation activates ULK1, which triggers autophagic processes that protect hepatocytes from I/R-induced damage. Inhibition of autophagy by blocking AMPK activation negates the protective effect of young plasma, confirming the pivotal role of autophagy in liver I/R protection (40). Thus, autophagy helps maintain cellular homeostasis, limits oxidative stress, and prevents the release of pro-apoptotic factors—thereby reducing hepatocyte injury at reperfusion (41–44). On the other hand, excessive or dysregulated autophagy can contribute to cell death in severe I/R conditions, amplifying tissue damage and inflammation. excessive autophagy is detrimental and has been shown to be induced by various factors during liver I/R. For example, both IL37 and Krüppel-like factor 6 (KLF6) have been linked to the regulation of autophagy in liver injury. IL37 overexpression, through suppression of key autophagy proteins (like LC3B II, Beclin1, and activation of p62), inhibits excessive autophagy during I/R and prevents hepatocyte apoptosis. Similarly, KLF6 inhibits the overactivation of autophagy by suppressing Beclin1 transcription, regulating the mTOR/ULK1 signaling axis. Overactivation of autophagy in the absence of KLF6 worsens liver damage, inflammation, and apoptosis (45, 46). These findings indicate that a finely balanced autophagic response is essential for hepatocyte survival in HIRI. Both the restoration of autophagy through AMPK/ULK1 activation and the inhibition of excessive autophagy through pathways like KLF6 and IL37 offer protective strategies against the pathological effects of HIRI.

## Mitophagy

Mitochondrial autophagy, or mitophagy, plays an essential role in maintaining mitochondrial quality by selectively degrading dysfunctional mitochondria, which is particularly critical in the context of HIRI where mitochondrial damage is extensive. In liver I/R, mitophagy facilitates the removal of mitochondria damaged by oxidative stress, thereby preventing the release of pro-apoptotic factors such as cytochrome c, and mitigating further cellular injury. Studies on mesenchymal stem cells (MSCs) and Parkin, a key protein in the mitophagy process, highlight the protective role of mitophagy in HIRI. In MSC-treated mice, mitophagy is upregulated, which leads to reduced mitochondrial reactive oxygen species (mtROS) production, improved mitochondrial function, and decreased hepatocellular apoptosis. Furthermore, the PINK1/Parkin pathway, which is crucial for initiating mitophagy, is found to be downregulated in I/R injury models but is restored by MSC treatment, further supporting the importance of mitophagy in protecting hepatocytes from I/R-induced damage (47, 48).

Mechanistically, the AMP-activated protein kinase (AMPK)/Unc-51-like kinase 1 (ULK1) signaling axis plays a central role in autophagy initiation. AMPK activation, often in response to energy depletion during ischemia, phosphorylates ULK1, thereby promoting autophagosome formation and subsequent mitophagy (49). This pathway ensures the clearance of damaged mitochondria and maintains cellular energy homeostasis. In HIRI, activation of the AMPK/ULK1 pathway has been associated with enhanced mitophagy and improved hepatocellular survival. However, autophagy’s role in HIRI is dual-faceted. While moderate autophagy is protective (50), excessive or dysregulated autophagy can be detrimental (51). Overactivation may lead to the degradation of essential cellular components, culminating in autophagic cell death. This paradox underscores the necessity for a balanced autophagic response during HIRI.

Mitochondrial dynamics and mitophagy are integral components of mitochondrial quality control, ensuring cellular homeostasis by regulating mitochondrial morphology and eliminating damaged mitochondria. These processes are particularly pertinent in the context of HIRI, where mitochondrial dysfunction plays a central role in hepatocellular damage (52). The interplay between mitochondrial dynamics and mitophagy is evident in the regulation of mitochondrial quality. Fission events often preceded mitophagy, isolating damaged mitochondrial fragments for removal. Disruptions in this interplay can lead to the accumulation of dysfunctional mitochondria, exacerbating cellular injury. Similarly, dysregulation of mitochondrial fusion and fission proteins to cardiovascular pathologies (53), highlighting the pivotal roles them, such as MFN1, MFN2, OPA1, and DRP1, in maintaining mitochondrial integrity and function (54). In HIRI, excessive mitochondrial fission and impaired mitophagy have been associated with increased hepatocellular apoptosis and necrosis (55). Mitochondrial dynamics encompass the continuous cycles of fusion and fission, mediated by proteins such as mitofusins (MFN1 and MFN2), optic atrophy 1 (OPA1), and dynamin-related protein 1 (DRP1). Fusion processes facilitate the mixing of mitochondrial contents, thereby diluting damaged components and maintaining mitochondrial function. Conversely, fission segregates impaired mitochondria, earmarking them for degradation via mitophagy (56).

Notably, several evidence indicates that the activity of PPIF (cyclophilin D) in regulating mitochondrial permeability transition pore (mPTP) opening has significant implications for autophagy in HIRI. When mPTP opening is triggered by Ca^2+^ overload and oxidative stress, it leads to a sudden drop in mitochondrial membrane potential that may overwhelm autophagic flux and accelerate hepatocyte death (57, 58). Conversely, inhibiting PPIF by pharmacological or genetic means—preserve mitochondrial integrity, allowing autophagy to fulfill its protective role of clearing only the most severely damaged mitochondria rather than precipitating a widespread necrotic or apoptotic response (15, 59).

## Ca^2+^ overload relief and PPIF mediated mPTP flickers

Calcium overload is a hallmark of ischemia-reperfusion (I/R) injury, playing a pivotal role in the initiation of mitochondrial permeability transition pore (mPTP) opening and catastrophic consequences for reperfused cells, such as necrosis and apoptosis (60, 61). Upon reperfusion, the rapid restoration of blood flow reactivates ATP-dependent ion pumps, which often fail due to mitochondrial dysfunction, leading to excessive calcium influx into the mitochondrial matrix. The accumulation of Ca^2+^ in mitochondria is known to trigger mPTP opening, which leads to mitochondrial depolarization, the collapse of oxidative phosphorylation, and ultimately, necrosis or apoptosis.

Traditional models have long positioned PPIF (cyclophilin D) as a principal culprit in hepatic ischemia-reperfusion injury, driving catastrophic opening of the mitochondrial permeability transition pore (mPTP), severe mitochondrial depolarization, and hepatocyte death. However, recent evidence—especially from studies in aging cells—suggests a protective dimension to PPIF activity under calcium overload conditions. Specifically, PPIF can facilitate intermittent or “flickering” openings of the mPTP, which serve to gradually release excessive Ca^2+^ from the mitochondrial matrix rather than allowing a single, prolonged pore opening (62). This flickering phenomenon helps maintain mitochondrial membrane potential and mitigates the collapse of oxidative phosphorylation, thereby preserving cell viability in the face of sustained Ca^2+^ stress. Intriguingly, complete knockout or pharmacological blockade of PPIF abrogates this controlled mPTP “flicker,” leading to heightened Ca^2+^ accumulation and decreased survival rates among aging cells (62).

In the context of hepatic I/R injury, hepatocytes often experience abrupt Ca^2+^ overload during the reperfusion phase when ATP-dependent pumps become reactivated but are still compromised by oxidative stress (63). The classical viewpoint holds that PPIF-mediated mPTP opening leads to uncontrolled loss of mitochondrial membrane potential, driving necrosis or apoptosis. The study on mPTP flickers highlighting that moderate or partial mPTP flickers—regulated by PPIF—can actually alleviate harmful Ca^2+^ buildup before it triggers large-scale pore openings. Thus, the net effect of PPIF on I/R outcome may reflect a balance between its capacity for short, protective pore flickers versus the risk of sustained pore openings. Under physiologic or moderately stressful conditions, limited PPIF activity may reduce Ca^2+^ toxicity and support hepatocellular survival; but when ischemic or oxidative insults are extreme, PPIF can tip the system into full-blown pore opening and cell death. Hence, total inhibition of PPIF might impede beneficial Ca^2+^ release events and exacerbate injury by allowing relentless Ca^2+^ overload, whereas unbridled PPIF activation risks catastrophic mPTP opening. Future therapeutic strategies for HIRI may thus aim to fine-tune PPIF activity—maintaining a controlled degree of mPTP flicker for Ca^2+^ homeostasis while preventing the pore from remaining fully open. This dualistic role underscores the complexity of PPIF’s function in HIRI, where both excessive and null PPIF expression can be detrimental, and highlights a pressing need for interventions that modulate, rather than merely block, mPTP dynamics.

## Controversies and limitations

Despite the well-established role of PPIF in promoting mitochondrial permeability transition pore (mPTP) opening, the exact molecular composition and regulatory mechanisms of the pore remain controversial. Early models centered on the adenine nucleotide translocase (ANT) as the main pore-forming component, while more recent studies implicate the c-ring of FoF1-ATP synthase (64, 22, 23). The precise molecular composition of the mitochondrial permeability transition pore (mPTP) remains a subject of ongoing debate, with several models proposed based on emerging experimental evidence (65). Alternatively, the adenine nucleotide translocator (ANT) has been implicated as a central component of the mPTP. Evidence indicates that ANT can form channels sensitive to calcium and bongkrekic acid, and its deletion alters mPTP characteristics. However, mPTP activity persists in ANT-deficient models, suggesting that ANT may function more as a regulatory element rather than the pore’s structural core (66). One prominent hypothesis posits that the c-subunit ring of the F_1_ F_0_ ATP synthase complex constitutes the core of the mPTP. Studies have demonstrated that purified c-subunit rings can form voltage-gated, high-conductance channels *in vitro*, and genetic ablation of the c-subunit significantly diminishes mPTP activity, underscoring its potential role in pore formation (67).

Moreover, heterogeneity among mitochondrial subpopulations contributes to another complexity. Investigations indicate that mitochondria within the same cell can differ in membrane potential, calcium-handling capacity, and susceptibility to pore opening, potentially explaining why some hepatocytes are more resistant to ischemia-reperfusion stress than others (68). It is also demonstrated that ischemia induces distinct morphological alterations across mitochondrial subpopulations in cardiomyocytes, such as increased sphericity and reduced length in intermyofibrillar mitochondria (69). These findings underscore the differential susceptibility and adaptive responses of mitochondrial subsets to ischemic conditions. Such heterogeneity highlights the need to refine our understanding of how PPIF regulates mPTP opening in distinct mitochondrial subtypes.

## Challenges in clinical translation

Although targeting PPIF/mPTP has yielded promising preclinical results, translating these findings into clinical settings remains challenging. Immunosuppressive properties of some cyclophilin inhibitors (e.g., cyclosporine A) or potential off-target effects pose safety and tolerability concerns. Additionally, delivering PPIF inhibitors or mitochondrial protectants specifically to hepatocytes while minimizing systemic toxicity is a key barrier. The zone-specific architecture of the liver and differential perfusion gradients can also affect drug distribution and therapeutic efficacy, highlighting a need for organ-specific or targeted-delivery strategies (70). Moreover, confounding variables—such as pre-existing liver disease, inflammation, or comorbid conditions—can alter drug metabolism and mitochondrial vulnerability, complicating outcome assessments in clinical trials.

Another critical yet unresolved issue is the potential risk associated with complete PPIF inhibition Although global knockout models of PPIF (CypD) demonstrate protection against ischemia-reperfusion injury, emerging evidence highlights that complete loss of PPIF may disrupt physiologically beneficial transient mPTP “flickering” openings. These intermittent pore openings are crucial in maintaining mitochondrial Ca²^+^ and ROS homeostasis, and their inhibition could paradoxically exacerbate mitochondrial dysfunction under certain conditions (62, 71). Moreover, the long-term implications of completely abolishing PPIF function, especially under varying metabolic stresses or chronic liver conditions, remain inadequately characterized, emphasizing the need for refined therapeutic approaches that preserve protective mPTP flickering while preventing catastrophic pore opening.

## Protective strategies targeting PPIF in HIRI

As noted, liver-specific deletion of the Ppif gene profoundly protects mice from I/R injury​ (25). This proof-of-concept suggests that any approach reducing PPIF expression or function in hepatocytes can be beneficial. While global PPIF knockout is not a practical therapy, transient knockdown via RNA interference or organ-specific gene therapy could be envisioned. Indeed, reducing CypD levels alleviates cell death by raising the threshold for mPTP opening. In parallel, genetic techniques that indirectly target mPTP have shown promise. Ischemia-tolerant mouse strains or those overexpressing anti-death proteins (like mitochondrial Bcl-2 family members) tend to keep mPTP closed longer, mimicking the PPIF-null phenotype. Encouragingly, CypD-knockout does not appear to harm normal liver function; mice lacking hepatic PPIF develop normally​ and have intact mitochondrial physiology under baseline conditions, indicating that temporary therapeutic suppression of PPIF is likely to be safe. These findings support exploring gene-silencing therapies or CRISPR-based approaches to transiently inhibit PPIF in patients at risk of I/R injury (such as prior to liver transplant or major liver resection).

Cyclophilin D inhibitors: Pharmacological inhibition of PPIF is a highly active area of research, with several classes of cyclophilin inhibitors tested in HIRI models. Cyclosporine A (CsA), a classic cyclophilin ligand, was one of the first agents shown to reduce mPTP-mediated cell death. CsA binds CypD in the mitochondrial matrix and blocks its PPIase activity, thereby preventing mPTP opening. In rat models of warm liver ischemia, CsA pretreatment significantly attenuated hepatocyte apoptosis and necrosis on reperfusion​ (25). However, CsA’s clinical use for I/R is complicated by its immunosuppressive effects (via cyclophilin A–calcineurin inhibition) and narrow therapeutic window. This has spurred development of non-immunosuppressive CsA analogs and novel CypD inhibitors. Alisporivir (Debio-025) and NIM811 are modified cyclosporine derivatives that retain cyclophilin binding but have reduced calcineurin affinity. These drugs have been shown to inhibit mPTP opening in experimental systems and were originally explored as antivirals and hepatoprotectants. In cell models of hepatic I/R, alisporivir was as effective as CsA at reducing LDH release and improving survival (28), confirming it can protect mitochondria from permeability transition. More recently, entirely new small-molecule cyclophilin inhibitors have emerged. Panel et al. identified several non-peptidic compounds (e.g. C31) that directly inhibit cyclophilin D’s peptidyl-prolyl isomerase activity​. These compounds prevented calcium-induced mitochondrial swelling *in vitro* and, when administered to mice, significantly reduced liver injury after I/R​ (16). Notably, some of these small molecules also exhibit CypD-independent effects on the mPTP​ (16), suggesting they may stabilize the pore or its components even in the absence of cyclophilin D – an added benefit for robustly preventing pore opening. Overall, pharmacological CypD inhibitors have proven to be powerful tools to blunt HIRI in preclinical studies, and they represent a tangible path toward therapy.

Mitochondria-targeted protective agents: Beyond direct Cyclophilin D inhibitors, various interventions that preserve mitochondrial integrity can indirectly modulate PPIF’s impact on HIRI. One strategy is to reduce the triggers of mPTP opening – namely calcium overload and oxidative stress – especially during the reperfusion phase. Ischemic postconditioning (brief cycles of reperfusion and re-occlusion applied at the onset of reperfusion) is one technique that has shown success. Postconditioning attenuates liver I/R injury in part by maintaining mitochondrial membrane potential and delaying mPTP opening; its benefits are lost if an mPTP opener (atractyloside) is applied, and are mimicked by exogenous CsA administration (72). This underscores that the protection from ischemic conditioning is largely via the PPIF/mPTP axis. Likewise, pharmacological agents that activate pro-survival signaling can inhibit mPTP through secondary pathways. Activation of the EP4 receptor (a prostaglandin E2 receptor subtype) was found to trigger an ERK1/2–GSK3β signaling cascade that ultimately keeps the mPTP closed, significantly reducing hepatocellular injury in I/R (73). GSK3β is known to phosphorylate and inhibit CypD, so EP4 agonists essentially reinforce an endogenous brake on PPIF, contributing to mitochondrial tolerance. Another avenue is mitochondria-targeted antioxidants. Compounds like MitoQ (mitoquinone) or XJB-5–131 concentrate in the mitochondrial membrane and scavenge ROS at the source. By limiting oxidative burst, they raise the threshold for mPTP opening (since oxidative damage to mitochondria is a key precipitant of CypD activation)​ (74). Experimental studies have shown that reducing mitochondrial ROS with such agents diminishes the release of DAMPs and inflammatory cytokines, thereby curbing the injury cascade. Melatonin, a potent antioxidant that accumulates in mitochondria, has also been reported to protect the liver from I/R injury through effects on the mPTP. Melatonin pretreatment in rats preserved mitochondrial membrane fluidity and prevented the loss of membrane potential during reperfusion​ (75). Mechanistically, melatonin attenuates the mitochondrial permeability transition after hepatic I/R, as evidenced by a decreased rate of mitochondrial swelling and cytochrome c release in treated livers​ (76). This suggests melatonin stabilizes mitochondrial membranes (possibly by interfering with CypD translocation or by reducing oxidative modifications of pore components), thereby inhibiting PPIF-mediated pore opening. Finally, enhancing mitochondrial quality control before I/R can mitigate PPIF’s detrimental effects. Interventions like ischemic preconditioning, or drugs that induce mild uncoupling, promote removal of damaged mitochondria (mitophagy) and improve the functional status of the organelles that face reperfusion. A healthier mitochondrial pool can better regulate calcium and ROS, indirectly restraining mPTP opening. In summary, a spectrum of protective strategies – from genetic knockdown and direct CypD inhibitors to signaling modulators and mitochondrial antioxidants – have shown efficacy in experimental models of HIRI. Common to all these strategies is the end result of a more tightly controlled (or inhibited) mPTP, underscoring PPIF as a hub for therapeutic intervention.

## Conclusion

Hepatic ischemia-reperfusion injury (HIRI) is a complex and multifaceted process that significantly impacts liver transplantation, hepatic surgeries, and the management of shock-induced liver failure. The mitochondria, as central players in cell death, mediate critical events in HIRI, particularly through the opening of the mitochondrial permeability transition pore (mPTP). Cyclophilin D (CypD), as a key regulator of mPTP opening, has emerged as a crucial mediator of mitochondrial dysfunction and hepatocyte injury during reperfusion. While excessive mPTP opening leads to cell death, recent studies suggest a protective role for PPIF (Cyclophilin D) in regulating intermittent mPTP openings, or “flickering,” which helps release calcium overload without triggering catastrophic mitochondrial collapse. This controlled mPTP flickering preserves mitochondrial integrity and supports hepatocyte survival under ischemic stress.

Additionally, autophagy, especially mitophagy, plays a crucial role in HIRI, balancing mitochondrial quality control and cellular damage. However, dysregulated autophagy can exacerbate liver injury, highlighting the need for precise modulation. Inhibiting excessive autophagy or enhancing autophagic activity through pathways such as AMPK/ULK1 offers promising protective strategies. PPIF’s involvement in both mitochondrial dysfunction and autophagy regulation makes it an attractive target for therapeutic interventions. Pharmacological inhibition of CypD, genetic knockdowns, and the development of new small molecules that modulate mPTP dynamics are showing promise in preclinical models and could represent potential therapeutic options to mitigate liver injury in HIRI. In summary, fine-tuning PPIF activity presents a promising therapeutic approach to improve outcomes in liver transplantation, liver resections, and other surgeries associated with ischemia-reperfusion injury.

Despite these advancements, significant translational challenges remain, including potential systemic side effects and off-target impacts associated with global PPIF inhibition, underscoring the necessity for targeted delivery methods. Specific areas of investigation should include the selective modulation of mPTP flickering to harness its protective physiological functions, thereby avoiding detrimental complete pore blockade. Organ-specific or targeted delivery of CypD inhibitors could enhance therapeutic specificity, reduce off-target effects, and improve clinical outcomes. Furthermore, combination therapies integrating modulation of mitochondrial dynamics (such as fusion and fission), mitophagy, and mitochondrial-targeted antioxidants might offer synergistic benefits. Such integrative therapeutic strategies hold promise for comprehensive mitochondrial quality control, effectively mitigating ischemia-reperfusion injury and improving patient outcomes following liver transplantation, hepatic resections, and other surgeries involving ischemia-reperfusion stress. In conclusion, finely tuned modulation of PPIF activity and mitochondrial quality control pathways emerges as a promising and critical strategy for future therapeutic development aimed at optimizing liver protection and recovery in clinical settings.
